# Fetal Electrocardiogram (fECG) Gated MRI

**DOI:** 10.3390/s130911271

**Published:** 2013-08-23

**Authors:** Martyn N.J. Paley, Janet E. Morris, Debbie Jarvis, Paul D. Griffiths

**Affiliations:** Academic Radiology, Department of Cardiovascular Science, University of Sheffield, Sheffield S10 2JF, UK; E-Mails: janet.morris@sth.nhs.uk (J.M.); deborah.jarvis@sth.nhs.uk (D.J.); p.griffiths@shef.ac.uk (P.G.)

**Keywords:** fetal electrocardiogram gating, magnetic resonance imaging, MRI safety

## Abstract

We have developed a Magnetic Resonance Imaging (MRI)-compatible system to enable gating of a scanner to the heartbeat of a foetus for cardiac, umbilical cord flow and other possible imaging applications. We performed radiofrequency safety testing prior to a fetal electrocardiogram (fECG) gated imaging study in pregnant volunteers (*n* = 3). A compact monitoring device with advanced software capable of reliably detecting both the maternal electrocardiogram (mECG) and fECG simultaneously was modified by the manufacturer (Monica Healthcare, Nottingham, UK) to provide an external TTL trigger signal from the detected fECG which could be used to trigger a standard 1.5 T MR (GE Healthcare, Milwaukee, WI, USA) gating system with suitable attenuation. The MR scanner was tested by triggering rapidly during image acquisition at a typical fetal heart rate (123 beats per minute) using a simulated fECG waveform fed into the gating system. Gated MR images were also acquired from volunteers who were attending for a repeat fetal Central Nervous System (CNS) examination using an additional rapid cardiac imaging sequence triggered from the measured fECG. No adverse safety effects were encountered. This is the first time fECG gating has been used with MRI and opens up a range of new possibilities to study a developing foetus.

## 1. Introduction

Triggering of *in utero* Magnetic Resonance Imaging (iuMRI) by the fetal ECG (fECG) would be useful for fetal cardiac studies or flow measurements in the umbilical cord. Currently no manufacturers provide the facility to gate to the fECG. The aim of this study was to perform MRI safety tests of a fECG sensor modified for MRI triggering capability together with a preliminary *in vivo* imaging feasibility study using the device on pregnant volunteers.

Real time imaging such as echo planar or single shot fast spin echo imaging can be used to effectively freeze fetal motion, but scans are not synchronized to the fetal cardiac cycle and generally have worse spatial resolution than can be achieved with gated acquisitions [1–3]. Self-gating, navigator-based methods avoid having to detect the fECG by acquiring a signal from the center of k-space with each k-space line acquisition, but have varying degrees of success dependent on patient motion and other factors [4–8]. Retrospective gating uses continuous acquisition of data together with labeling of each acquisition of the cardiac phase at the time of acquisition, but this is only possible with an fECG signal. The data is then retrospectively binned and reconstructed resulting in a more efficient acquisition strategy and ensuring the data is in steady state at all times throughout the acquisition. Metric-optimized gated imaging is a variant on retrospective gating where the ECG signal cannot be measured directly. The data are acquired continually over periods longer than the anticipated heart beat interval and then assigned to various bins according to a model of the fetal heart rate. The ghosting errors which arise in the reconstructed data are then minimized using a measure such as time-entropy through an iterative procedure by moving acquired k-space lines between the different cardiac cycle intervals [9,10].

Ultrasound is an established method for looking at flow in the foetus, placenta and umbilical cord providing non-invasive high resolution images with the possible of Doppler velocity imaging and the potential to gate an MRI scanner. However, ultrasound can have problems due to acoustic shadowing from, for example, bone, which can be a problem in cases with low amniotic fluid levels, maternal obesity or with difficult fetal positioning within the uterus. In addition quantitative ultrasound flow measurements rely on the shape of the vessels, the insonation angle and the velocity profile which are difficult to quantify in a single study [11].

A modified cardiac triggering device has previously been used to examine the fetal sheep heart [12] but in this paper we attempt to use the directly detected human fetal ECG to obviate many of the difficulties discussed above and gain access to a full range of gated imaging sequences in terms of image contrast and acquisition speed. Combined with maternal breath hold, fECG gating should allow cardiac imaging and phase contrast velocity mapping to be acquired from the foetus and umbilical cord in future.

## 2. Experimental Section

**Figure 1.**
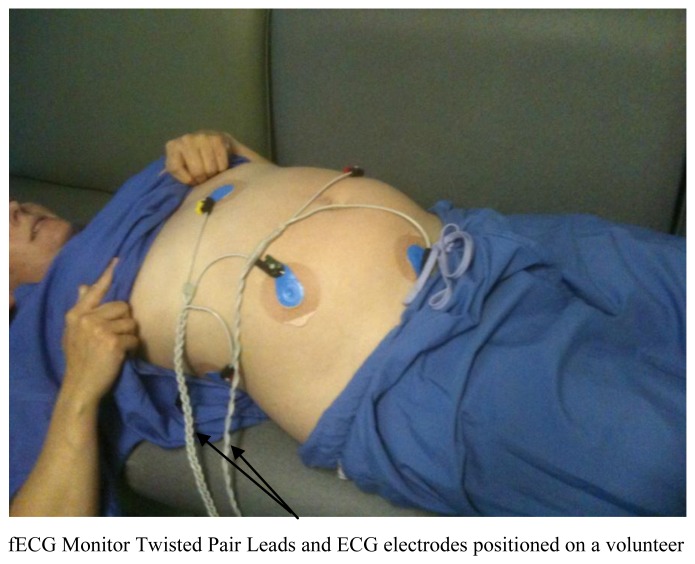
Placement of the ECG electrodes on a pregnant volunteer. The electrodes used were Ambu Blue Sensor VLC single patient use ECG electrodes (Ambu A/S, Ballerup, Denmark) with a skin contact diameter of 68 mm. The skin was gently abraded using a dermal abrasive tape (3 M, Bracknell, UK) prior to locating the electrodes.

**Figure 2.**
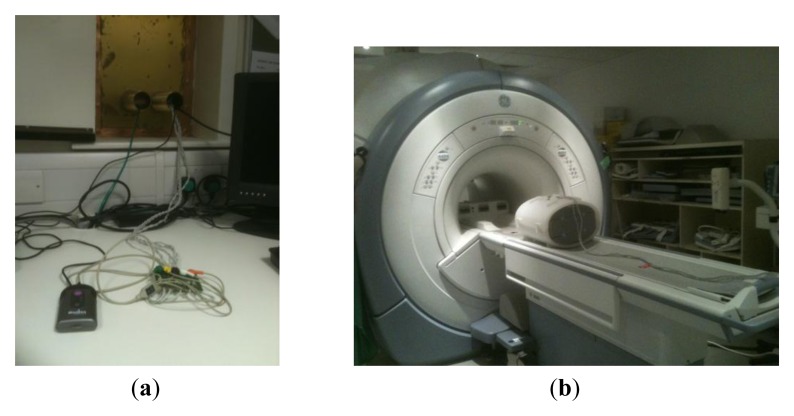
(**a**) The portable fetal and maternal ECG used for the study (AN24, Monica Healthcare, Nottingham, UK). The device records the data into internal memory over long periods but also transmits the maternal and fetal ECG data (four channels) in real time over a Bluetooth interface. The fECG monitor was connected to the six metre MR compatible high resistance leads which go through a waveguide into the scan room. The five leads were twisted as a pair and as a triplet. The trigger signal from the device was attenuated by 30 db and fed back into the scan room using a BNC cable (unfiltered in this case, although filtering could be used in future); (**b**) The leads attached to the MR compatible electrodes positioned on a torso phantom on a bed of conducting gel prior to location within the magnet for confirmatory safety tests.

A compact monitoring device (Figure 1) with advanced software capable of reliably detecting both the maternal ECG (mECG) and fECG traces simultaneously was modified by the manufacturer (Model AN24, Monica Healthcare, Nottingham, UK) to provide an external TTL trigger signal from the detected fECG signal. Six meter long high resistance carbon ECG leads were fed through waveguides into the MR scan room from the device (Figure 2a). The leads were attached to MR compatible electrodes and placed on a large torso shaped imaging test object in locations similar to those which would be used for fetal gating. The surface of the test object was covered with conducting gel as used for the electrodes to provide a circuit for any possible RF pickup (Figure 2b). A single shot fast spin echo (SSFSE) sequence was run with the same imaging parameters as used for clinical in-utero scanning using the body transmit coil. The radiofrequency voltage generated at 64 MHz was measured across the leads using a 100 MHz input bandwidth digital oscilloscope (Tektronix, Beaverton, OR, USA).

A waveform file provided by the device manufacturer produced a voltage using the sound card of a laptop computer to simulate the mECG and fECG signals. To test gating performance, the fECG signal measured through the MR compatible leads was converted into a digital gating pulse which was attenuated by 30 db and fed back into the standard MR ECG system. A balanced steady state gradient echo FIESTA sequence with SLT = 5 mm, in-plane resolution = 2 mm, NEX = 1, TR/TE = 3.3/1.4 ms was acquired with fECG gating at 123 bpm.

This work was performed with the approval of and under the guidance of the NHS Research Ethics Service (reference 10/H1308). All women provided written informed consent prior to their studies. Pregnant women (*n* = 3) with known Central Nervous System (CNS) pathology of their foetus coming for a follow up examination (so they already had experience of an MRI examination) were assessed using the fetal ECG system. The leads were attached to five MR compatible electrodes and placed on the lower abdomen close to the anticipated location of the fetal heart prior to all scanning and presence of the fetal trigger pulse checked after patient positioning in the magnet. Images were acquired using a 1.5 T HDx MR System (GE Healthcare, Milwaukee, WI, USA) using all the sequences included in our standard in-utero CNS imaging protocol plus an additional fECG gated cardiac cine sequence at the end of the examination using the same geometry as planned for the CNS examination to comply with granted ethical permission. Typical overall in magnet time was 30 min.

## 3. Results and Discussion

Radiofrequency voltages at 63.9 MHz measured across the leads were always significantly less than ±1 V peak to peak using the single shot fast spin echo sequence set up for a 70 kg load which is much lower than would produce a radiofrequency burn. The electrodes and test object were relocated within the RF body coil four times to assess reproducibility and the same result was observed each time. Images were acquired with the electrodes in place using a gradient echo sequence and there was no evidence of RF or B_0_ interference.

**Figure 3.**
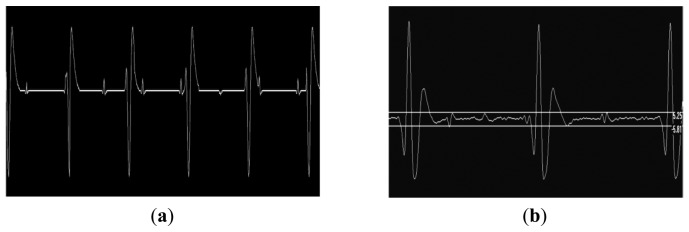
(**a**) Five maternal cycles of the simulated maternal and fetal ecg waveform used for the gating tests. This waveform was played from a PC soundcard and attenuated to the peak level expected for maternal *in vivo* signals (100 μV); (**b**) Two cycles of the signal received over the Bluetooth interface using the six metre, high resistance MR compatible leads showing the additional noise introduced. The measured ‘fetal’ signal from the simulator measured about 10 μV.

**Figure 4.**
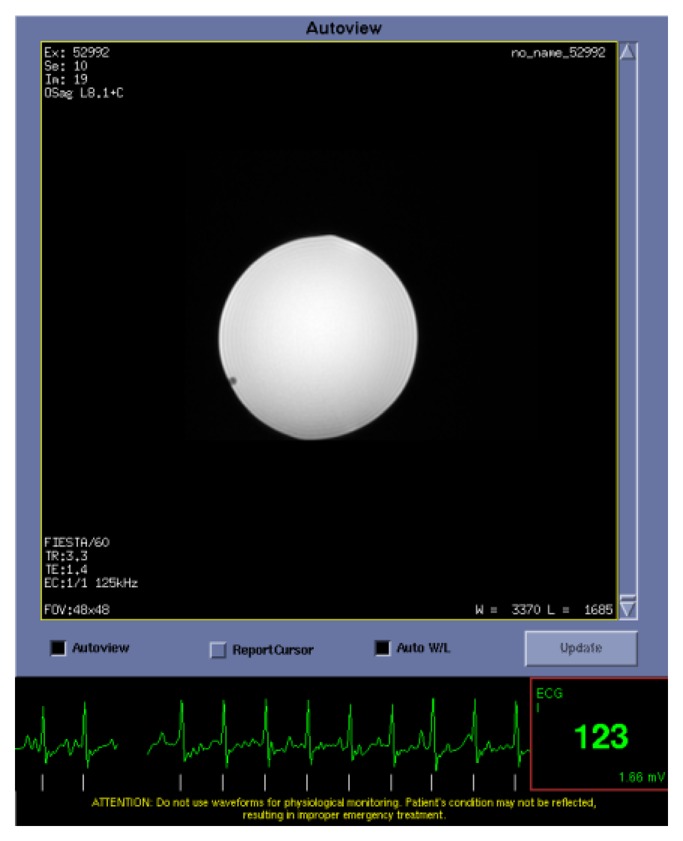
A FIESTA sequence with TR = 3.3 ms, TR = 1.4 ms, NEX = 1, in-plane spatial resolution = 1 × 1 mm, SLT = 5 mm acquired with the cardiac gating signal from the simulated fetal ECG at 123 beats per minute acquired over the 6 m MR compatible leads. The images show no artifacts from the leads or electrodes.

The fECG and mECG signals could be measured in real time using the Bluetooth interface (Figure 3). Occasionally, when the fetal and maternal ECG signals coincided, the fetal gating signal was lost due to software checks within the device which excluded it during this time. Test object images were acquired at the fetal heart rate (123 bpm) to demonstrate the rapid gating capability of the MR system (Figure 4).

**Figure 5.**
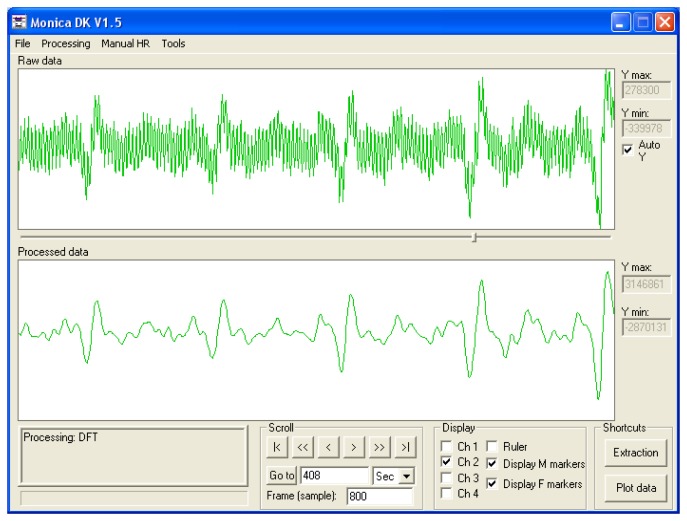
The effects of Finite Impulse Response (FIR) filtering with a standard bandpass filter (4–45 Hz) on the combined mECG and fECG signals which were acquired from a volunteer within the magnet (without imaging applied). ECG data was recorded during the entire imaging session and subsequently downloaded from the device and analysed using a developmental software tool from the monitor manufacturer DK V1.5 (Monica Healthcare, Nottingham, UK).

Following satisfactory safety testing on test objects, the volunteer study was initiated. No safety issues were encountered using the device to record the fECG during fetal imaging on the pregnant volunteers. The gating system was present for all imaging sequences. Figure 5 shows raw and Finite Impulse Response (FIR)-filtered (4 Hz–45 Hz bandpass provided with the Monica Healthcare software) ECG data acquired in the magnet without imaging gradients present clearly showing mECG and fECG components at different rates. The mECG was approximately 80 bpm and the fECG was approximately 140 bpm for all three volunteers and foetuses.

**Figure 6.**
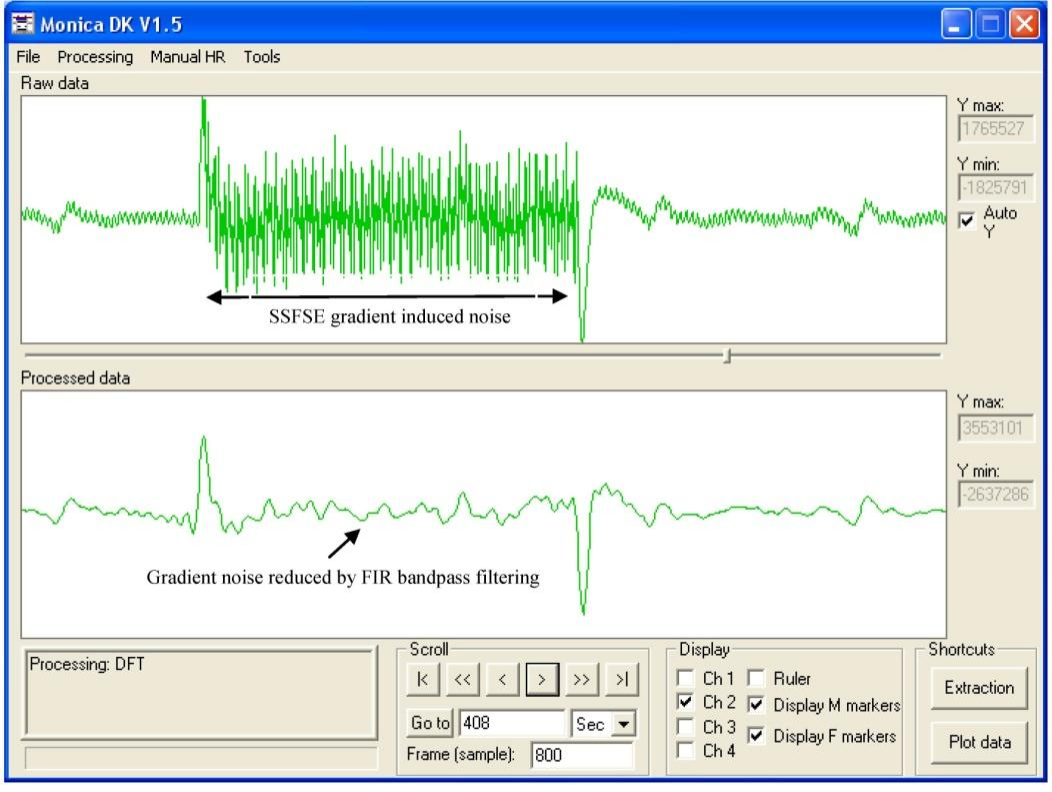
The effects of a single shot fast spin echo sequence gradient burst on the fECG trace (upper plot). The effects of a bandpass FIR filter are shown in the lower trace. The effects of the gradient pulses are largely removed from the trace but the phase of the ECG signal is temporarily altered.

Figure 6 illustrates the effects of burst of gradient waveforms from a single shot spin echo sequence on the fECG trace. Filtering, as contained in the fECG hardware largely removes the effects of this rapid train of pulses.

**Figure 7.**
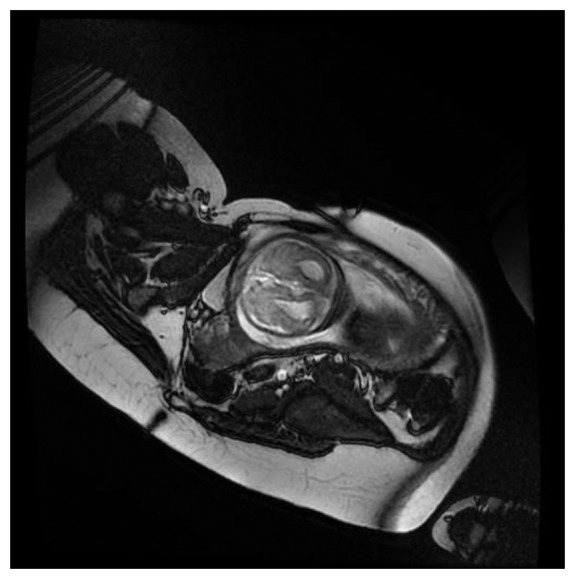
A fECG gated rapid image acquired from a foetus in an oblique plane from one of the pregnant volunteers. As this feasibility study was performed at the end of a fetal CNS pathology examination, it was not possible to specifically plan and visualise the heart under ethical guidance. None of the volunteers reported any adverse effects during imaging.

Figure 7 show a fECG gated fetal image from one of the volunteers at the end of the normal CNS exam using a gradient echo cardiac gated imaging sequence (Right Ventricle Long Axis (RVLA) sequence) but using the geometry as planned for the CNS examination.

## 4. Conclusions/Outlook

Availability of a reliable fetal ECG gating signal should open up a wealth of new opportunities for non-invasively measuring the fetal cardio-vascular system. Although self-gating techniques are reasonably successful in detecting the cardiac cycle, other MR related issues can modify the detected self-gating signal such as approach to steady state, contrast preparation as well as maternal and/or fetal bulk motion which can limit the applicability and accuracy.

In this study it has been demonstrated for the first time that it is possible to safely gate an MR scanner to the fECG using a modified fECG monitor and carbon fibre lead system. It was also possible to observe the mECG and fECG signals in real-time on a linked computer display using a Bluetooth interface available on the fECG device. The fECG signals reliably triggered a fast MR image acquisition sequence at the fetal heart rate from the simulated signal at 123 bpm. No interference between the gating system and the MR system was observed in terms of generated noise lines on the images in these preliminary studies.

Some gradient interference on the gating signal was observed which occasionally resulted in mis-triggering if the fetal gating signal occurred during gradient activity. This was minimized by using a short duration data acquisition relative to the fetal ECG period. To improve reliability of fetal triggering for longer duration data acquisitions such as used for single shot fast spin echo, improved real-time filtering of the ECG signal as demonstrated in Figure 6 could be included in the device firmware. In addition, locating the device in the scan room and shortening the twisted pair leads would also improve sensitivity and reliability. The device was kept out of the magnet room and long leads were used in this study as the device contained magnetic components which could have lead to a projectile hazard and also contained components sensitive to the effects of the RF pulses generated by the MR scanner.

Fetal cardiac gated images were acquired from three pregnant volunteers showing the capability of the fetal monitor to operate successfully within the MR environment using extended leads. This should provide new opportunities for examining the developing cardiovascular system and umbilical blood supply gated to the fetal rather than maternal circulation. This should also allow development of quantitative flow protocols through gated velocity phase mapping sequences.
